# A Comprehensive Proteomic Survey of ABA-Induced Protein Phosphorylation in Rice (*Oryza sativa* L.)

**DOI:** 10.3390/ijms18010060

**Published:** 2017-01-03

**Authors:** Jiehua Qiu, Yuxuan Hou, Yifeng Wang, Zhiyong Li, Juan Zhao, Xiaohong Tong, Haiyan Lin, Xiangjin Wei, Hejun Ao, Jian Zhang

**Affiliations:** 1 State Key Lab of Rice Biology, China National Rice Research Institute, Hangzhou 311400, China; qjh3636@sina.com (J.Q.); houyuxuan@caas.cn (Y.H.); wangyifeng@caas.cn (Y.W.); lzhy1418@163.com (Z.L.); zhaojuan521321@163.com (J.Z.); tongxiaohong@caas.cn (X.T.); linhaiyan@188.com (H.L.); weixiangjin@caas.cn (X.W.); 2 Agricultural Genomes Institute at Shenzhen, Chinese Academy of Agricultural Sciences, Shenzhen 518120, China; 3 College of Agricultural Sciences, Hunan Agricultural University, Changsha 410128, China; aohejun@126.com

**Keywords:** rice (*Oryza sativa* L.), abscisic acid, signaling, phosphoproteome

## Abstract

abscisic acid (ABA) is a key phytohormone regulating plant development and stress response. The signal transduction of ABA largely relies on protein phosphorylation. However; little is known about the phosphorylation events occurring during ABA signaling in rice thus far. By employing a label-free; MS (Mass Spectrometry)-based phosphoproteomic approach; we identified 2271 phosphosites of young rice seedlings and their intensity dynamics in response to ABA; during which 1060 proteins were found to be differentially phosphorylated. Western-blot analysis verified the differential phosphorylation pattern of D1, SMG1 and SAPK9 as indicated by the MS result; suggesting the high reliability of our phosphoproteomic data. The DP (differentially phosphorylated) proteins are extensively involved in ABA as well as other hormone signaling pathways. It is suggested that ABA antagonistically regulates brassinosteroid (BR) signaling via inhibiting BR receptor activity. The result of this study not only expanded our knowledge of rice phosphoproteome, but also shed more light on the pattern of protein phosphorylation in ABA signaling.

## 1. Introduction

Abscisic acid (ABA) is an isoprenoid hormone playing critical roles in various plant biological processes. Limited cellular water availability is a major abiotic factor leading to ABA accumulation and signal transduction [1]. Under dehydration or other types of osmotic stress, ABA promotes the stomata closure in guard cells to maintain water homeostasis in plants. The desiccation during seed maturation also induces high levels of ABA accumulation, which leads to seed dormancy and germination inhibition [2]. ABA signaling is a broad topic consisting of ABA metabolism and transport, ABA perception and signal transduction, as well as the ABA signal response and modulation [1]. Based on knowledge from Arabidopsis, a new “PYR/PYL/RCAR-PP2C-SnRK2” cascade model for ABA signaling has been proposed and validated [3,4]. The three core components of this model play vital but different roles in ABA signaling. PYR (PYrabactin Resistance 1)/PYL (PYR1-like)/RCAR (Regulatory Components of ABA Receptor) are water soluble ligand-binding proteins belonging to START (star-related lipid-transfer)-domain superfamily, and have been shown to be ABA receptors by two independent research groups [5,6]. PYR/PYL/RCAR harbors binding sites for PP2Cs (2C-type protein phosphatase), which are negative regulators in ABA signaling. SnRK2s (SNF1-related protein kinases 2) are plant specific serine/threonine protein kinases bridging the ABA signal transduction from PP2Cs to the downstream ABA-responsive element (ABRE)-binding protein as well as other downstream components [7]. In the absence of or under low levels of ABA, PP2Cs physically bind with and dephosphorylate SnRK2s to inhibit their kinase activity, which finally results in the silence of the ABA signaling pathway. In contrast, upon the signal perception of ABA accumulation induced by stresses, PYR/PYL/RCAR competitively bind with PP2Cs to release the SnRK2s from PP2Cs. Subsequently, autophosphorylation of the released SnRK2s activates their own kinase activity and transmits the ABA signal to downstream ARFs, and thereby promotes the ABA or stress responses [3,8].

Given that ABA signal transmission largely relies on protein phosphorylation/dephosphorylation, profiling the phosphorylation substrates and sites of the core proteins in the ABA signaling pathway has attracted great interests in the community. In Arabidopsis, an in vitro kinase assay revealed that PP2C type phosphatase ABI1 (Abscisic acid insensitive 1) and HAB1 (Hypersensitive to ABA 1) dephosphorylate the SnRK2.6/OST1 protein at the Ser175 in the absence of ABA. Meanwhile, Ser175 is also a critical site for the SnRK2.6/OST1 autophosphorylation when the PP2C inhibition is removed [4]. Among the 10 members of the SnRK2 protein family in Arabidopsis, SnRK2.2/SRK2D, SnRK2.3/SRK2I and OST1/SnRK2.6 are most actively involved in ABA signaling, and many of their substrate proteins have been successfully identified in the past decades [9,10,11]. Sirichandra et al. [12] (2009) found that OST1 phosphorylates Ser13 and Ser174 of AtrbohD, a key enzyme for reactive oxygen species production in guard cells, to control stomatal closure [13]. A substitution of Ser174 into Alanine significantly reduces the phosphorylation of AtrbohF by OST1 [12]. OST1 also determines the stomatal pore aperture by phosphorylating the K^+^ channel protein KAT1. It was shown that Thr306 is the key phosphosite regulating KAT1’s activity [14]. ABA-responsive-element Binding Factor 3 (ABF3), which is a bZIP transcription factor controlling part of the ABA-regulated transcriptome, was also found to be a genuine OST1 substrate. OST1 preferentially phosphorylates ABF3 on multiple LxRxxpS/T motifs including Ser126 and Thr451, but also weakly phosphorylates other sites such as Ser32 and Ser134 [15]. In addition, some other proteins such as SLAC1 (Slow Anion Channel-associated 1), ICE1 (Inducer of CBF expression 1) and QUAC1 (Quickly activating Anion Channel 1) have been reported to be OST1 phosphorylation substrates, although their phosphosites were not clearly indicated [16,17,18,19]. Besides OST1, SnRK2.2 and SnRK2.3 have ABA-inducible kinase activity as well, the synergism of both are likely to play a major role in phosphorylating and activating downstream substrates such as ABF2, ABI5 and ABF1 [11,20,21]. Furthermore, the phosphorylation sites of these substrates were identified by various methods. For example, at least eight sites of ABI5 (ABA Insensitive 5), including Thr35, Ser36, Ser41, Ser42, Ser138, Ser139, Ser145 and Thr201, were found to be phosphorylated by MS analysis [22]. In rice, there are 10 SnRK2 family members which are named SAPK1-10 (osmotic Stress/ABA-activated Protein Kinase) [23]. Only SAPK6, SAPK8, SAPK9 and SAPK10 show ABA-inducible patterns, indicating that they are involved in ABA signaling [24,25]. SAPK8-10 are able to phosphorylate TRAB1, a key regulator mediating ABA-induced transcription, at the region of amino acid 91–171. Further evidence also showed that at least Ser94 and Ser102 of TRAB1 were phosphorylated by SAPK10 [25]. By using a MALDI-TOF (Matrix-Assisted Laser Desorption/Ionization Time of Flight) analysis, Chae et al. [24] (2007) identified Ser44 of OREB1 as a critical phosphosite of OSRK1/SAPK6, and mutation of the residue greatly decreased the substrate specificity for OSRK1.

Despite many efforts as mentioned above, the majority of the known ABA-induced phosphoproteins were identified through traditional biochemical kinase assays which are technically challenging and time-consuming, and therefore make our knowledge in this field rather limited and fragmented. Recently, development of MS-based proteomic approaches have allowed scientists to quantitatively identify the ABA-related protein phosphorylation events in a large scale. Kelin et al. [26] (2010) identified 50 Arabidopsis phosphopeptides which were differentially phosphorylated between control and ABA treated samples. One hundred and fifty-two phosphopeptides were differentially phosphorylated after treatment with five different phytohormones including ABA [27]. In an attempt to identify the SnRK2 protein kinase substrates in Arabidopsis, two independent labs conducted phosphoproteomic experiments in *snrk2.2*/*2.3*/*2.6* triple mutants as well as the wild type [28,29]. Umezawa et al. [28] (2013) identified 35 peptides, including AtMPK1, AtMPK2, AREB1 and SNS1, which were specifically phosphorylated in Wild-type but not in the triple mutant plants treated with ABA. Similarly, Wang et al. [29] (2013) found that 58 proteins were not sensitive or less sensitive to ABA treatment in the triple mutants. Further in vitro kinase assays confirmed that most of the 58 proteins were SnRK2 substrates involved in various biological processes. In addition to the SnRK2 deficient plants, a paper was published very recently reporting the rapid phosphoproteomic effects of ABA on wild type and ABA receptor quadruple mutants in Arabidopsis [30].

Rice is a major crop feeding over half of the global population. It has been fully recognized that ABA plays critical roles in rice seed germination, grain filling, biotic and abiotic stress responses [31,32]. Therefore, clarifying the molecular mechanism of ABA signaling in rice would be of great biological as well as economic significance for rice genetic improvement. Unfortunately, the protein phosphorylation events, in particular the phosphosites and intensity dynamics, in ABA signaling have been barely studied. In this study, we focused on the profiling of young rice seedling phosphosites, phosphopeptides and phosphorylation intensity dynamics in response to ABA by employing a label-free, MS-based phosphoproteomic approach, and aimed to reveal the biological significance of protein phosphorylation in ABA signaling from the phosphoproteomic data.

## 2. Results

### 2.1. Profile of the Abscisic Acid (ABA)-Induced Phosphoproteome in Rice

**Figure 1:**
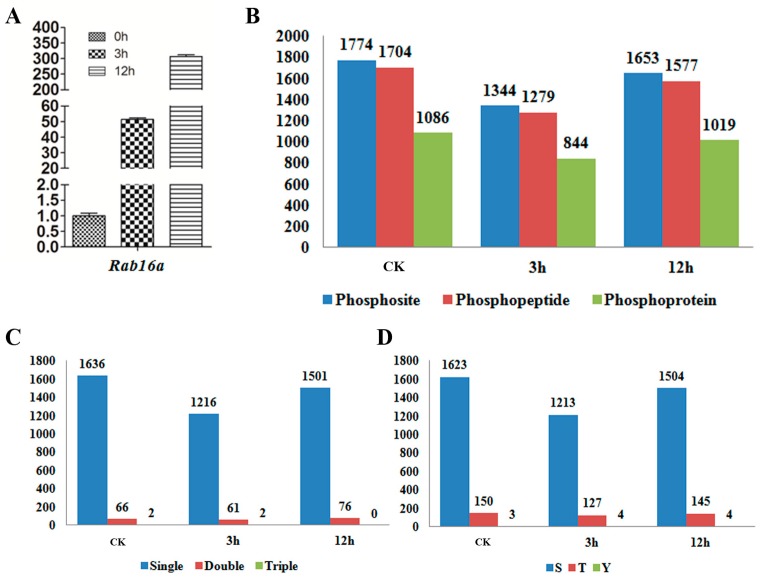
(**A**) qRT-PCR (quantitative Real Time-Polymerase Chain Reaction) analysis of abscisic acid (ABA) responsive gene *Rab16a* in CK, 3 and 12 h samples; (**B**) The number of identified phosphosites, phosphopeptides and phosphoproteins; (**C**) The counts of phosphopeptides carrying single, double and triple phosphorylation modifications; (**D**) The counts of phosphosites in serine, threonine and tyrosine.

Profiling the phosphorylation proteins, peptides and sites is fundamental and crucial to understand the roles of protein phosphorylation in ABA signaling in rice. In the current study, a MS-based, quantitative, label-free phosphoproteomic approach was employed to identify the phosphopeptides and phosphosites from 14-day-old young seedlings of Nipponbare (*Oryza sativa*, ssp. *japonica*) upon ABA treatment. Because exogenous ABA could be efficiently up taken by intact rice roots [33], the ABA treatment was conducted by immersing young seedlings in water containing 100 µM ABA. Previous study indicated that robust rice proteomic response to exogenous ABA started at 3 h and lasted over 6 h [34], we therefore chose 3 and 12 h to represent the ABA early and late signaling stages respectively, while seedlings without ABA treatment was used as a control (CK). Samples in each time point were conducted with three biological replicates. Prior to the protein extraction for MS analysis, we tested the transcriptional level of *Rab16a* (LOC_Os11g26790), a marker gene of ABA induction in rice, by quantitative RT-PCR (Real Time Polymerase Chain Reaction) [35]. As shown in Figure 1A, the level of *Rab16a* was significantly increased in response to ABA, indicating a valid ABA treatment in the experiment. In this study, a total of 1774, 1344 and 1653 phosphosites from 1704, 1279 and 1577 phosphopeptdies were identified in CK, 3 and 12 h, respectively (Figure 1B). After putative redundancy was filtered and removed, collectively 2271 phosphosites on 2162 phosphopeptides were profiled on 1086 phosphoproteins. Over 90% of the phosphopeptides in all the three samples carried only one phosphorylation modification, close to 10% of the phosphopeptides were phosphorylated twice, whereas triple phosphorylation was very rarely detected (Figure 1C). Additionally, we counted ratios phosphorylation which occurred on serine, threonine and tyrosine respectively. Phosphoserine was the predominant phosphorylations type and accounted for over 94% of the phosphosites, around 5% of the phosphosites were on threonine, and only 0.2%–0.3% of the phosphosites were on tyrosine (Figure 1D).

### 2.2. Differentially Phosphorylated Proteins in Response to ABA Treatment

**Figure 2:**
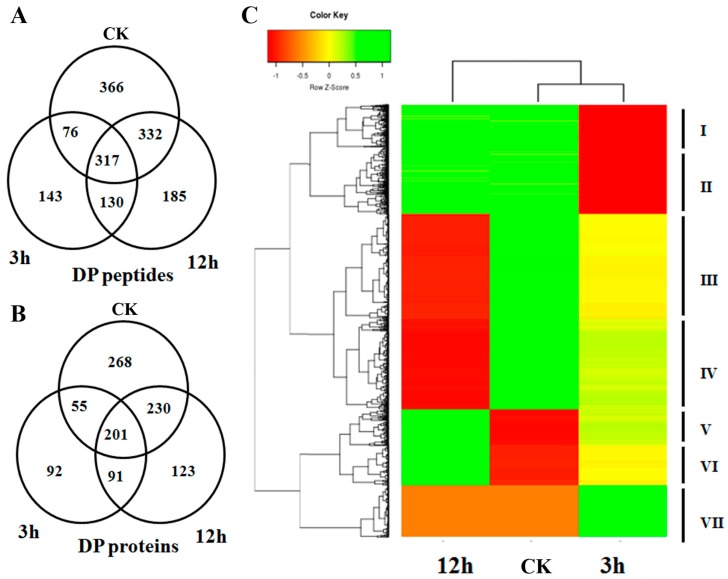
(**A**) Venn diagram showing the number of differentially phosphorylated (DP) phosphopeptides identified in control (CK), 3 and 12 h; (**B**) Venn diagram showing the number of DP phosphoproteins identified in CK, 3 and 12 h; (**C**) Hierarchical clustering analysis of the DP proteins among CK, 3 and 12 h. Color bar on the left represents the log2 phosphorylation intensity values. Red, yellow and green indicate the low, medium and high phosphorylation intensity values respectively.

A differential phosphorylation pattern usually indicates the important regulatory roles of the phosphoprotein in the corresponding biological process. Upon ABA treatment, a total of 1549 peptides were found to be differentially phosphorylated (Fold change > 2, *p* < 0.05). There were 366, 143 and 185 peptides which were specifically phosphorylated in CK, 3 and 12 h respectively. Meanwhile, 130, 332 and 76 peptides were found to be specifically not phosphorylated in CK, 3 and 12 h (Figure 2A). At the protein level, we identified 1060 DP proteins, including 268, 92, 123 proteins which were specifically phosphorylated and 91, 230, 55 proteins which were specifically not phosphorylated, in CK, 3 and 12 h respectively (Figure 2B). Among the DP proteins, we found 96 kinases and 19 phosphatases whose function are specifically for protein phosphorylation or dephosphorylation. In addition, the DP proteins also covered 65 transcription factors, including 9 bZIP family members (Table S1). It is known that bZIP is a major group of ABA responsive factors that could be phosphorylated by SnRK2 kinases in ABA signaling. The ABA-induced phosphorylation pattern strongly implied that these bZIPs are functionally involved in ABA signaling. By blasting against the potential SnRK2 target proteins obtained from Arabidopsis [28,29], we found that 154 of the DP proteins are close orthologs of these SnRK2 targets (*E* value < 1 × 10^−10^, score ≥ 80 and identity ≥ 80), suggesting their involvement in the ABA core signaling pathway (Table S2). Based on the phosphorylation intensity pattern at the three time points, a hierarchical clustering analysis divided the DP proteins into seven clades, indicating their divergent roles in ABA signaling (Figure 2C; Table S3). For example, proteins in clade V had a low level of phosphorylation at CK, while the phosphorylation intensity increased at 3 h and reached the maximum number at 12 h, indicating their roles in ABA response in the late stage. Meanwhile, proteins in clade VII are proposed to be involved in the early ABA signaling because their phosphorylation intensity significantly elevated at 3 h, but soon dropped back to a low level at 12 h. In addition, the co-phosphorylation pattern of the proteins in each clade hinted that the members may work in the same or similar pathways of ABA signaling.

### 2.3. Motif-X and GO (Gene Ontology) Analysis of Differentially Phosphorylated Proteins

**Figure 3:**
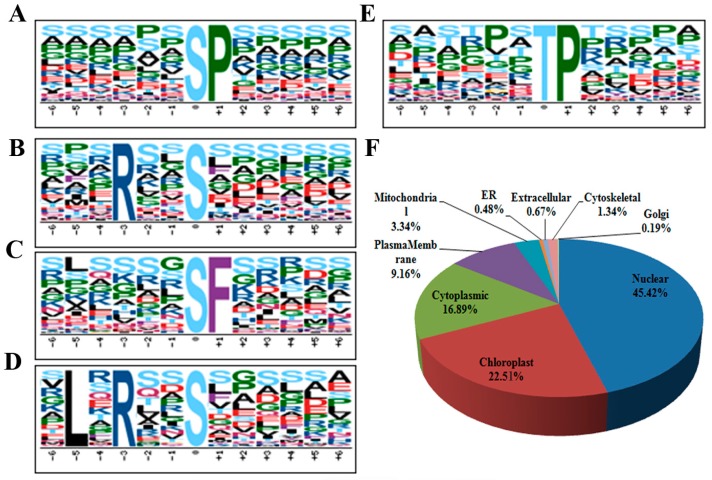
Motif-X analysis of the over-represented motifs around the phosphosites of the differentially phosphorylated (DP) proteins in response to ABA treatment. (**A**) (sP); (**B**) (Rxxs); (**C**) (sF); (**D**) (LxRxxs); (**E**) (tP); (**F**) Distribution of the DP phosphoproteins in subcellular compartments.

Substrate specificity of kinases is largely determined by the motifs harboring the phosphosite. Therefore, a view of the conserved motifs around phosphosites of the DP proteins may provide useful clues to find the kinases catalyzing phosphorylation on them. With the aid of a written program, we extracted a 13 amino acid-long sequence with the phosphosite in the center for all the 1636 unique phosphosites on DP proteins, including 1484 phosphoserines, 148 phosphothreonines and 4 phosphotyrosines. Subsequently, 1589 AA sequences were extracted and inputted into an online tool Motif-X (http://motif-x.med.harvard.edu/) [36] for conserved motif detection and resulted in the identification of 4 phosphoserine motifs (sequences number *n* > 100) and 1 phosphothreonine motif (*n* > 100) (Figure 3A–E). As found in many other cases, proline-directed motifs (sP) and (tP) are most commonly present around the phosphosites of our DP proteins [37,38,39,40]. Proteins with a (sP) motif have been reported to be substrates of MAPKs, SnRK2s (sucrose non-fermenting1-related protein kinase 2), RLKs (receptor-like kinases) and many other kinases [41]. We also identified two basophilic motifs (Rxxs) and (LxRxxs) which could be recognized by MAPKK, CaMK (calmodulin-dependent protein kinase)-II and protein kinase A [41]. Interestingly, (sF) has been rarely reported by previous reports in many species including rice [37,38,41]; this study together with two of our previous cases we found it as an over-represented motif in rice leaf, seed and seedlings [39,40]. This observation might be ascribed to the difference in methods of protein extraction and MS identification.

By using the protein subcellular localization prediction tool WoLF PSORT (http://www.genscript.com/wolf-psort.html) [42], we analyzed protein distribution in various cellular compartments. The results showed that nuclear, chloroplast and cytoplasmic are the three most enriched cellular compartments which accounted for 45.4%, 22.5% and 16.9% of the DP protein destinations. It was also found that 9.2% and 3.3% of the DP proteins are localized in the plasma membrane and mitochondria respectively, while less than 3% of the DP proteins are distributed in the remaining six cellular compartments analyzed (Figure 3F). It has been known that ABA signal activates a lot of ion channels and transporters, which are usually lipid-soluble proteins located in plasma membrane. The detection of relatively less number of plasma membrane proteins may attribute to the phenol method used for protein preparation, which majorly extracted the water-soluble proteins but excluded some of the lipid-soluble proteins. Therefore, phosphoproteomic analysis of proteins isolated from various cellular fractions may be necessary to obtain a more comprehensive result.

**Figure 4:**
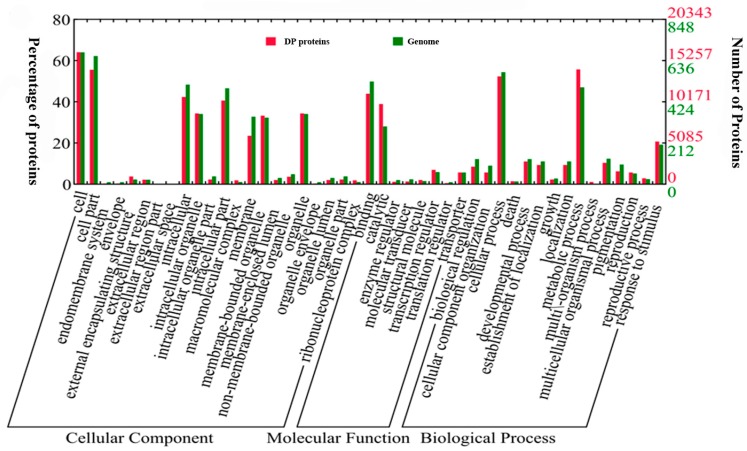
GO (Gene Ontology) analysis of DP proteins in terms of cellular components, molecular functions and biological processes.

To assign the biological relevance of the DP proteins, GO (Gene Ontology) analysis of them was performed in terms of “cellular component”, “molecular function” and “biological process” and visualized by WEGO (Web Gene Ontology Annotation Plot) [43]. In the perspective of “cellular component”, categories of “cell” and “external encapsulating structure” were enriched, while “cell part”, “intracellular” and “membrane” were under-represented when compared with the distribution ratio of the rice whole genome encoding proteins (Figure 4). Interestingly, we found that the ABA DP proteins showed over-represented molecular functions in “catalytic”. Given that proteins in “catalytic” are mostly enzymes like kinases and phosphatases, the enriched distribution suggested the extensive involvement of kinases and phosphatases in the ABA response. On the contrary, the “binding” was less favored for the ABA-induced DP proteins (Figure 4). In terms of “biological process”, DP proteins were enriched in “metabolic process” and “response to stimuli”, suggesting that ABA predominantly affects the signaling and metabolism via protein phosphorylation. Meanwhile, we found that DP proteins were less preferentially distributed in “cellular process” and “cellular component organization”.

### 2.4. Validation of the Phosphorylation Patterns by Western-Blot

**Figure 5:**
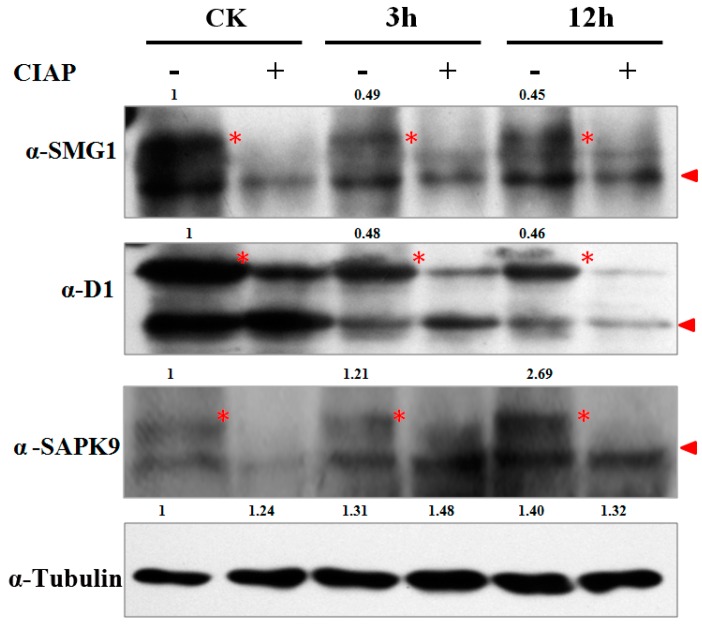
Western-blot analysis of SMG1, D1 and SAPK9 to verify the Mass Spectrometry (MS)-identified phosphorylation pattern at CK, 3 and 12 h. Red triangle indicates the target band in original size; Red asterisk indicates the phosphorylated target protein band. Anti-tubulin was used as an internal control for normalization. The values above the phosphorylated bands represent the normalized, relative band intensities by setting CK to 1.

To validate the MS identified phosphorylation sites of the DP proteins, we manually checked the spectrum map of D1 (LOC_Os05g26890), SMG1 (LOC_Os02g54600) and SAPK9 (LOC_Os12g39630) (Figure S1) and synthesized their antibodies for Western-blot analysis. Because phosphorylation could increase the molecular weight and lag the target protein band shift in electrophoresis, it is expected to have a bigger band on the top of the original protein band if protein phosphorylation has occurred. Samples were also treated with CIAP (Calf Intestine Alkaline Phosphatase) to confirm that the lagged band is the phosphorylated target protein, instead of a non-specific background band in the Western-blot. β-Tubulin was used as an internal control for the semi-quantification of signals in each sample. The western-blot results showed that the relative phosphorylation band signals of SMG1 at 3 and 12 h were decreased to 49% and 45% respectively when CK was used as the control, which is in consensus to the 43% and 66% in our phosphoproteomic data (Figure 5, Table 1). The result of D1 was similar to SMG1. The MS identification revealed that SAPK9 was lowly phosphorylated at CK, but slowly increased phosphorylation at 3 and 12 h. Accordingly, we found that the phosphorylation band signals of SAPK9 at CK, 3 and 12 h were 1, 1.21 and 2.69 respectively (Figure 5, Table 1). The results above strongly suggested that our phosphoproteomic data is highly reliable and such a MS-based quantification strategy could be applied for the phosphorylation dynamic detections in other biological processes.

## 3. Discussion

In plants, it has been widely accepted that ABA signals are transmitted majorly through the “PYR/PYL/RCAR-PP2C-SnRK2” cascade, in which protein phosphorylation/dephosphorylation plays essential roles [3]. As the first step toward understanding ABA signaling, profiling the ABA-induced phosphoproteins, phosphosites and phosphorylation dynamics is fundamental. So far, most of our knowledge on this issue was obtained from fragmented kinase function characterization researches by using traditional biochemical assays. Some primary phosphoproteomic methods such as 2D-DIGE (two dimension difference gel electrophoresis) were applied for the investigation of phosphorylation patterns in rice upon ABA treatment, but yielded limitedphosphoproteins data [34,44]. Compared with 2D-DIGE, high resolution LC-MS (liquid chromatograph-mass spectrometer)/MS has been proven to be more sensitive, efficient and accurate in phosphoprotein identification. In the current study, we demonstrated a high through-put, accurate, phosphoproteomic identification of the ABA-induced protein phosphorylation events in rice by utilizing a label-free, MS-based approach. A total of 1086, 844 and 1019 phosphoproteins were profiled from young rice seedlings upon ABA treatment at three time points, representing the largest dataset of ABA-induced phosphoproteins in plants. In addition, our MS data provided highly reliable phosphorylation intensity quantification, which has been verified by the Western-blot analysis of SMG1, D1 and SAPK9. With the quantification data, 1060 phosphoproteins including kinases, phosphotases, transcription factors and so on, were found to be differentially phosphorylated in response to ABA application, suggesting that ABA signaling is a highly complex process with a wide range of proteins involved. Meanwhile, the DP pattern also revealed the potential roles of these proteins in ABA response.

According to the “PYR/PYL/RCAR-PP2C-SnRK2” model, protein phosphorylation starts from the SnRK2s’ autophosphorylation, while no phosphorylation would occur on ABA receptors and PP2Cs (more specifically, the clade A PP2Cs) as they only competitively bind with ABA or each other to decide the phosphorylation status of SnRK2s [3]. The rice genome contains 12 ABA receptors, 10 clade A *PP2Cs* and 10 *SnRK2* genes [23,45,46]. Consistent with previous reports, we did not find any ABA receptors or clade A PP2Cs in our DP protein list. On the contrary, the phosphorylation of 2 SnRK2 including SAPK6 and 9 were significantly induced by ABA treatment. Kobayashi et al. [23] (2004) demonstrated that SAPK8, 9 and 10 protein accumulations is induced by ABA treatment. There is also literatures suggesting that ABA up-regulate the transcription levels of SAPK4 and 6, but the final effects in the protein level remain unclear [24,47]. Assuming that an induced expression pattern usually indicates the function in the process, it is rationale for us to propose that SAPK6, 8, 9 and 10 are functionally involved in the ABA signaling. Nevertheless, our study did not detect any obvious phosphorylation change in SAPK8 and 10. To explain this discrepancy, we checked the mRNA expression level of SAPK8 and 10 in different tissues, and found that the expression of both genes in young seedlings is in extremely low in abundance, which may result in the poor or omitted MS identification of them. Other possible reasons could be that SAPK8 and 10 respond to ABA in an earlier or later stage which is beyond the range checked in this study, or the growth conditions, such as high humidity (90%) during hydroponic culture down-regulated the ABA signaling pathway prior to the ABA treatment. Therefore, developing more sensitive MS identification equipments and methods, and also extending the time range of ABA treatment will be helpful for us to thoroughly profile the ABA induced phosphorylation events.

Phytohormone regulation highly complex process, in which the reception of one hormone usually triggers the synergistic or agonistic interplay with other hormones. It has been known that brassinosteroids (BRs) act antagonistically with ABA in promoting seed germination and post-germinative growth. The BR signaling repressor BIN2 (Brassinosteroid INsensitive 2) can stabilize and phosphorylate the positive ABA regulator ABI5 (Abscisic Acid Insensitive 5) in presence of ABA, thus positively regulate ABA response. The antagonism from BR to ABA is probably mediated by the inactivation of the BIN2-ABI5 cascade when BRs are applied to the plants [48]. However, a view of the ABA-BR antagonism from the ABA side has not yet been achieved. In the current study, we found that SERK2/OsBAK1 (LOC_Os04g38480), an ortholog of Arabidopsis BR co-receptor BAK1 (BRI1-Associated receptor Kinase 1) was down-phosphorylated by ABA treatment. In Arabidopsis, the BAK1-BRI1 complex binds with BR to sense the signal [49]. Suppressing of *SERK2* in rice leads to severe BR insensitivity and other BR-deficient phenotypes such as altered plant architecture and immune response, indicating SERK2 plays key roles in BR signaling, possibly as a receptor [50,51]. The ABA-induced dephosphorylation of SERK2 provided strong evidence that ABA antagonistically regulates BR signaling via inhibiting the BR receptor activity. In addition to BR components, several proteins involved in the signaling of other phytohormones were differentially phosphorylated as well. For example, D1/RGA1 (LOC_Os05g26890) is a key regulator of the G protein-dependent GA signal transduction pathway. *d1* plants show pleiotropic phenotypes including dwarf, erected panicles, small and roundish seed shape due to the reduced cell proliferation [52]. Our phosphoproteomic data revealed that the phosphorylation intensities of D1/RGA1 at 3 and 12 h were decreased to 30% and 48% respectively when compared with that of the CK, which is in agreement with the ABA-GA antagonistic effects. Moreover, MHZ7 (LOC_Os07g06130) involved in ethylene signaling, OsDOS (LOC_Os01g09620) and C3H12 (LOC_Os01g68860) participating in JA (jasmonic acid) signaling were all differentially phosphorylated during the ABA treatment, suggesting the extensive cross-talk between ABA and these phytohormones [53,54].

Downstream of the constructed “PYR/PYL/RCAR-PP2C-SnRK2” ABA signaling pathway, the ABA signal is finally transmitted by prtoein phosphorylation from SnRK2 to ABF transcription factors, of which many have been reported to be bZIPs [25,55,56,57]. In the current study, we detected 10 differentially phosphorylated bZIPs, 6 of which showed an up-phosphorylation pattern upon ABA induction. bZIP72 is a member of the group A OsbZIP family with ABRE trans activity in yeast [55]. qRT-PCR analysis showed that *bZIP72* was highly expressed in most of the rice tissues and can be induced by ABA, ethylene, MeJA, PEG, salinity and cold. More interestingly, *bZIP72* over-expression lines became hypersensitive to ABA, and showed retarded seedling growth, delayed seed germination and enhanced drought tolerance when compared with the Wild-type. *ABF1* (*ABA responsive* *element binding factor 1*) is another reported ABA signaling-related *bZIP* gene. The expression of *ABF1* is induced by ABA and various abiotic stresses. In the allelic mutants *Osabf1-1* and *Osabf1-2*, some ABA responsive genes were suppressed, and the plants became more sensitive to drought and salinity treatments than wild type plants [57]. Though it has been known that ABA induces the quantity of *bZIP72* and *ABF1* in the mRNA level, how these two bZIPs are involved in ABA signaling remains unclear. Their DP pattern as revealed in this study may suggest that bZIP72 and ABF1 participate in the ABA signaling via the phosphorylation activation from the upstream components in the pathway, possibly SnRK2s. More importantly, the revealed phosphosites of these transcription factors provided valuable information for studies to elucidate the mechanism of these proteins in ABA signaling. For example, bZIP72 harbors over 40 potential phosphosites as predicted by Rice Phospho 1.0 tool [58]. However, only the Ser71 and Ser116 of bZIP72 were detected and up-phosphrylated under ABA treatment in our phosphoproteomic data (Table 1). Thus, Ser71 and Ser116 are very likely to be the target sites of bZIP72 in ABA signaling, which will be further investigated in our lab in the future.

## 4. Methods

### 4.1. ABA Treatment

Fourteen-day-old Nipponbare young seedlings were obtained from pure water culture in a growth chambers (28 °C, 90% relative humidity, 14 h daytime and 10 h night time). For the ABA treatment, healthy, intact young seedlings were kept in a beaker with water containing 100 µM ABA (Sigma, St. Louis, MO, USA). The whole seedling samples were collected at CK, 3 and 12 h respectively, and immediately used for RNA isolation and protein extraction.

### 4.2. RNA Isolation and qRT-PCR (Quantitative Real Time-Polymerase Chain Reaction)

The total RNA of the whole young seedlings was extracted by Trizol (Invitrogen, Carlsbad, CA, USA) according to the manufacturer’s instructions. DNaseI (Takara, Dalian, China) was added in the total RNA for eliminating DNA contamination. The DNA free total RNA was quantified by a Nanodrop spectrometer (Thermo, Waltham, MA, USA) and 2 µg RNA was used for each reverse transcription by using M-MLV reverse transcriptase (Takara, Dalian, China) with Oligo(dT)_20_ (Invitrogen, Carlsbad, CA, USA) as primer. Quantitative real-time RT-PCR analysis was conducted in a total volume of 10 µL containing 2 µL reverse-transcribed product above, 0.2 µM each primer and 5 µL THUNDERBIRD SYBR^®^ qPCR Mix (Toyobo, Shanghai, China) on a CFX96 touch realtime PCR detection system (Bio-Rad, Hercules, CA, USA). The *ubiquitin* (GenBank accession No. AF184280) was used as an internal control. The relative expression level of tested genes was calculated based on *C*_t_ values by following the 2^−ΔΔ*C*t^ method[59]. Primers sequences are as follows: Ubif: 5′-GCTCCGTGGCGGTATCAT-3′; Ubir: 5′-CGGCAGTTGACAGCCCTAG-3′; Rab16af: 5′-GAGGGAGGAGCACAAGACC-3′ and Rab16ar: 5′-ATTCCATCATCCTCAGAC GAG-3′.

### 4.3. Protein Extraction

Three grams of each sample was ground into fine powder in liquid nitrogen, homogenized well in 15 mL PEB buffer (0.9 M Sucrose, 0.5 M Tris-HCl, 0.05 M EDTA, 0.1 M KCl, 2% β-Mercaptoethanol, pH = 8.7), extracted with same volume of saturated phenol (pH = 4.2) by shaking on ice for 30 min. The extracted proteins in the phenol phase were precipitated in 5 volumes of ice-cold PS (Precipitation Solution) buffer (0.1 M Ammonium Acetate, 1% β-Mercaptoethanol, in 100% Methanol), washed in 75% ethanol for three times and finally dissolved in PBS (Phosphate Buffer Saline) buffer (137 mM NaCl, 2.7 mM KCl, 10 mM Na_2_HPO_4_, 2 mM KH_2_PO_4_).

### 4.4. Protein Digestion and Phosphopeptide Enrichment

The procedures were done as described by Qiu et al. [40] (2016). Briefly, protein was treated for a sequential of 5 mM DTT (DL-Dithiothreitol) reduction, 20 mM IAA (indole-3-acetic acid) alkylation and again DTT reduction in the dark. The treated proteins were digested on the 30 kDa filter unit (Millipore, Darmstadt, Germany) over night with trypsin at pH = 8.0 (enzyme:protein = 1:50). After the filter-aided sample preparation (FASP), peptides were desalted using C18 Sep-Pak (Waters, Milford, MA, USA) and redissolved in binding buffer (80% ACN, 5% TFA, 1 M laccaic acid). Subsequently, peptides were incubated with TiO_2_ beads (GL sciences, Tokyo, Japan) with a ratio of peptide:TiO_2_ = 1:4 for 3 × 30 min, washed with binding buffer, and then eluted with the elution buffer (40% ACN, 15% NH_3_H_2_O) 4 times. Eluates were subsequently vacuum dried and reconstituted with 5% MeOH in 1% C solution for LC-MS/MS analysis.

### 4.5. LC-MS/MS (Liquid Chromatograph-Mass Spectrometer/Mass Spectrometer) and Data Analysis

This was done by following the methods of Qiu et al. [40] (2016) in Beijing Proteome Research Center. LC-MS/MS analyses were performed on an Easy-nLC 1000 liquid chromatography system (Thermo, Waltham, WA, USA) coupled to a Q-Exactive Plus via a nano-electrospray ion source (Thermo). The peptide mixture was eluted from a 360-µm ID × 2 cm, C18 trap column and separated on a homemade 100 µm ID × 10 cm column (C18, 1.9 µm, 120 Å, Maisch GmbH) with a linear 5%–35% acetonitrile gradient at 500 nL/min. Survey scan were acquired after accumulation of 8.06 × 10^2^ ions in Orbitrap for *m*/*z* 300–1400 using a resolution of 70,000 at *m*/*z* 400. The top 20 intense precursor ions were selected for fragmentation in the HCD cell at normalized collision energy of 27%, and then fragment ions were transferred into the Orbitrap analyzer operating at a resolution of 17,500 at *m*/*z* 400. The dynamic exclusion of previously acquired precursor ions was enabled at 18 s. Raw spectral data were processed for phosphopeptide identification and phosphosite quantification in Proteome Discoverer 1.4.1.14 suites with Mascot search engine against the rice genome annotation project database: (ftp://ftp.plantbiology.msu.edu/pub/data/Eukaryotic_Projects/o_sativa/annotation_dbs/pseudomolecules/version_7.0/all.dir/). The mass tolerance was set at 20 ppm for precursor, and 50 mmu for the tolerance of product ions. Oxidation (M), Acetyl (Protein-N term) and Phospho (S/T/Y) were set as variable modifications, and Carbamidomethyl (C) as static modification in the Mascot searches for phosphopeptides. Two missed cleavage on trypsin was allowed. The results were filtered for peptide at high identification confidence (False discovery rates < 1%) and *E* Value < 0.05 by the Percolator tool of the Protein Discoverer package. Low-scoring peptides (Mascot score ≤ 20) were excluded from the analysis when they were not further supported by additional high-scoring identifications in other replicates or experiments. All phosphopeptide hits were automatically re-analyzed by the phosphoRS software within the Protein Discoverer software suite. PhosphoRS probability higher than 90% was required for a phosphorylation site to be considered as localized. Only those peptides which were phosphorylated in at least two of the three biological replicates were considered as truly phosphorylated. The phosphorylation is quantified based on the peak area under the ion intensity by using precursor ions area detector in PD1.4.1.14. Extracted ion chromatogram (sum of areas under the curves of MS1 chromatograms of each peptide identified) was used for normalization across all runs. Within-group means were calculated to determine fold changes. The differentially phosphorylated protein was defined to have over two fold changes in the average intensity with credible student’s *t*-test (*p* < 0.05). The mass spectrometry proteomics data have been deposited to the ProteomeXchange Consortium [60] via the PRIDE partner repository with the dataset identifier PXD004939.

### 4.6. Western-Blot Analysis

To dephosphorylate the proteins, each 20 µg protein sample was treated by 1 µL of calf intestinal alkaline phosphatase (Takara, Dalian, China) at 37 °C for 2 h. Western-blot analysis was performed as described by Qiu et al. [40] 2016. Briefly, 20 µg denatured total protein was resolved in 10% SDS (sodium dodecyl sulfate)-polyacrylamide gels. Then, the separated proteins bands were transferred onto a 0.45 µm polyvinylidene fluoride fluoropolymer (PVDF) membrane (Millpore, Darmstadt, Germany) by using an electrophoretic blotting system (Bio-Rad, Hercules, CA, USA). The target protein bands were sequentially detected with corresponding primary antibodies (1:1000 dilution), secondary antibody IgG conjugated with a HRP (Horseradish Peroxidase) (1:10,000 dilution), and visualized by using the enhanced chemiluminescence (Pierce, Waltham, WA, USA). Anti-D1, anti-SAPK9 and anti-SMG1 were synthesized by Genescript Company (Shanghai, China), while anti-β-tubulin (Cat No. M20005, Abmart, Berkeley, CA, USA), anti-GST (Cat: CW0085, CWBIO, Beijing, China) and anti-His (Cat: CW0083, CWBIO, Beijing, China) were commercially purchased. Quantification of the band intensities on the immune-blots was performed using the ImageJ software (v2.1.4.7) according to the instructions (http://rsb.info.nih.gov/ij/docs/menus/analyze.html#gels). All the sample intensities were first normalized to the control tubulin, and then calculated based on the ratio to set the relative level of CK into 1.

## 5. Conclusions

Here, we report the profiling of 2271 phosphosites on 2162 phosphopeptides from young rice seedlings and the quantification of their intensity dynamics in response to ABA. 1060 proteins were found to be differentially phosphorylated during the ABA treatment. The DP proteins are extensively involved in ABA as well as other hormone signaling pathways. It is suggested that ABA antagonistically regulates BR signaling via inhibiting the BR receptor activity. Western-blot analysis verified the differential phosphorylation pattern of D1, SMG1 and SAPK9 as indicated by the MS result. Our result of this study expands our knowledge of rice phosphoproteome, but also provides insight into the mechanism of protein phosphorylation in ABA signaling.

## Figures and Tables

**Table 1:** Some selected examples of the differentially phosphorylated (DP) proteins identified under abscisic acid (ABA) treatment.

Sequence	Protein Group	Annotation	Abbreviation	Modifications	Average CK	Average 3 h	Average 12 h
VLPAQQSSPR	LOC_Os01g09620	Zinc finger/CCCH transcription factor	OsDOS	S8 (Phospho)	0	0	4.09 × 10^8^
GGGGSAGLGSMNVEEILR	LOC_Os01g64730	bZIP transcription factor	OsABF1	S5 (Phospho); S10 (Phospho)	0	5.15 × 10^7^	4.27 × 10^7^
LQSPGAQQTYGTSQQVDASAGNQGMLSPYR	LOC_Os01g68860	Zinc finger C-x8-C-x5-C-x3-H type family protein	C3H12	S3 (Phospho)	7.78 × 10^7^	0	0
STVGTPAYIAPEVLSR	LOC_Os02g34600	CAMK_CAMK_like.13	SAPK6	T2 (Phospho)	0	3.31 × 10^7^	0
AGLQQQQQQQPGTPGR	LOC_Os02g54600	STE_MEK_ste7_MAP2K.5—STE kinases	SMG1	T13 (Phospho)	5.30 × 10^8^	2.31 × 10^8^	3.50 × 10^8^
VQAHQGSASFR	LOC_Os03g16570	Zinc finger, C3HC4 type domain containing protein	OsSDIR1	S9 (Phospho)	3.39 × 10^7^	4.57 × 10^7^	0
HNDWIVDSTYNLR	LOC_Os04g38480	BRASSINOSTEROID INSENSITIVE 1-associated receptor kinase 1 precursor	OsSERK2	S8 (Phospho)	6.81 × 10^7^	0	0
AMELSGPR	LOC_Os04g38480	BRASSINOSTEROID INSENSITIVE 1-associated receptor kinase 1 precursor	OsSERK2	S5 (Phospho)	6.81 × 10^7^	0	0
EVLSSEPEEIGNDEK	LOC_Os04g55230	Tetratricopeptide repeat domain containing protein	FLO-2	S5 (Phospho)	3.82 × 10^7^	0	0
YVISPDNQEIGEK	LOC_Os05g26890	G-protein alpha subunit	D1/RGA1	S4 (Phospho)	1.82 × 10^7^	8.12 × 10^6^	1.21 × 10^7^
DNLQGSAFLGSSR	LOC_Os07g06130	Ethylene-insensitive protein	MHZ7	S12 (Phospho)	0	2.52 × 10^7^	7.02 × 10^7^
HPFFAVSAPASPTR	LOC_Os07g39220	BES1/BZR1 homolog protein	OsBZR1	S7 (Phospho); S11 (Phospho)	6.49 × 10^7^	0	0
ADSPNPSSGDHPAGVGGSPEK	LOC_Os07g39480	OsWRKY78	OsWRKY78	S18 (Phospho)	3.04 × 10^7^	0	0
DFGSMNMDELLR	LOC_Os09g28310	bZIP transcription factor	bZIP72	S4 (Phospho)	0	0	1.48 × 10^7^
QGSLTLPR	LOC_Os09g28310	bZIP transcription factor	bZIP72	S3 (Phospho)	6.65 × 10^7^	1.83 × 10^8^	3.15 × 10^8^
STVGTPAYIAPEVLLK	LOC_Os12g39630	CAMK_CAMK_like.49	SAPK9	T2 (Phospho)	0	6.51 × 10^7^	9.71 × 10^7^

## Supplementary Materials

Supplementary materials can be found at www.mdpi.com/1422-0067/18/1/60/s1.
