# Supplementary material for: A Comprehensive Proteomic Survey of ABA-Induced Protein Phosphorylation in Rice (Oryza sativa L.)
Source: Int J Mol Sci. 2017 Jan 3;18(1):60. doi: 10.3390/ijms18010060 (PMC5297695; doi:10.3390/ijms18010060)
Supplement: Supplementary file 1 [file ijms-18-00060-s001.zip › ijms-162935-suppl .pdf]

Supplementary Materials: A Comprehensive Proteomic Survey of ABA-Induced Protein Phosphorylation in Rice (*Oryza sativa* L.)

Jiehua Qiu, Yuxuan Hou, Yifeng Wang, Zhiyong Li, Juan Zhao, Xiaohong Tong, Haiyan Lin, Xiangjin Wei, Hejun Ao and Jian Zhang

A

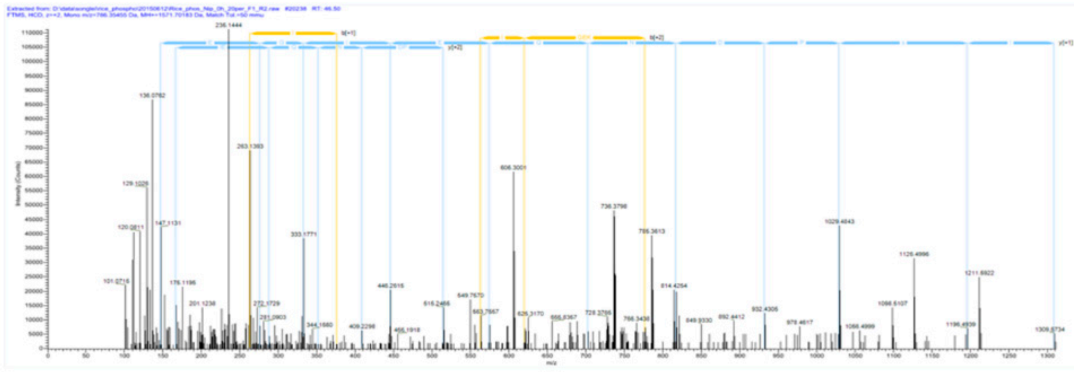

Y V I s P D N Q E I G E K

D1/RGA1

B

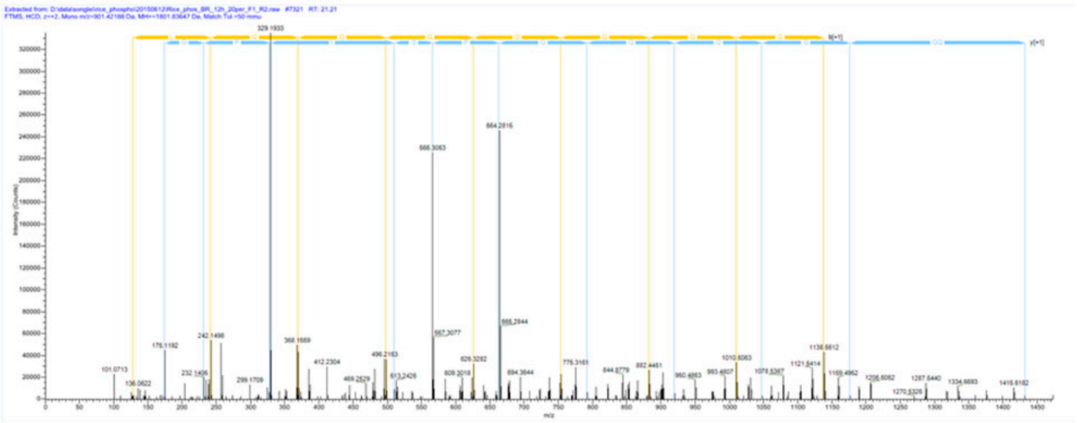

A G L Q Q Q Q Q Q Q P G t P G R

SMG1

Figure S1. The spectrum map of some selected phosphoproteins. (**A**) The spectrum map of protein D1; (**B**) The spectrum map of protein SMG1, Yellow and blue represent the b and y fragments respectively.
